# DNA aptamer selection and construction of an aptasensor based on graphene FETs for Zika virus NS1 protein detection

**DOI:** 10.3762/bjnano.13.78

**Published:** 2022-09-02

**Authors:** Nathalie B F Almeida, Thiago A S L Sousa, Viviane C F Santos, Camila M S Lacerda, Thais G Silva, Rafaella F Q Grenfell, Flavio Plentz, Antero S R Andrade

**Affiliations:** 1 Centro de Desenvolvimento da Tecnologia Nuclear (CDTN), Avenida Presidente Antônio Carlos 6627, Belo Horizonte, CEP 31270-901, Brazil; 2 Departamento de Física, ICEx, Universidade Federal de Minas Gerais, Avenida Presidente Antônio Carlos 6627, Belo Horizonte, CEP 31270-901, Brazil; 3 MedicOnChip, Parque Tecnológico de Belo Horizonte-BH-TEC, Rua Professor José Vieira de Mendonça 770, Belo Horizonte, CEP 31310-260, Brazil; 4 Current address: DTU Physics, Technical University of Denmark, Kongens Lyngby, 2800, Denmark; 5 Instituto René Rachou - Fundação Oswaldo Cruz, Avenida Augusto de Lima 1715, Belo Horizonte, Minas Gerais, 30190-002, Brazil

**Keywords:** aptamer, biosensor, graphene, NS1 protein, Zika virus

## Abstract

Zika virus (ZIKV) is a mosquito-borne virus that is phylogenetically close to other medically important flaviviruses with high global public health significance, such as dengue (DENV) and yellow fever (YFV) viruses. Correct diagnosis of a flavivirus infection can be challenging, particularly in world regions where more than one flavivirus co-circulates and YFV vaccination is mandatory. Acid nucleic aptamers are oligonucleotides that bind to a specific target molecule with high affinity and specificity. Because of their unique characteristics, aptamers are promising tools for biosensor development. Aptamers are usually obtained through a procedure called “systematic evolution of ligands by exponential enrichment” (SELEX). In this study, we select an aptamer (termed ZIKV60) by capillary electrophoresis SELEX (CE-SELEX) to the Zika virus non-structural protein 1 (NS1) and counterselection against the NS1 proteins of DENV (serotypes 1, 2, 3, and 4) and YFV. The ZIKV60 dissociation constant (*K*_d_) is determined by enzyme-linked oligonucleotide assay (ELONA) and the aptamer specificity is evaluated by quantitative real-time polymerase chain reaction. ZIKV60 shows a high binding affinity to the ZIKV NS1 protein with a *K*_d_ value of 2.28 ± 0.28 nM. The aptamer presents high specificity for ZIKV NS1 compared to NS1 of DENV and YFV. Furthermore, graphene field-effect transistor devices functionalized with ZIKV60 exhibit an evident identification of NS1 protein diluted in human serum. These results point to the applicability of biosensors based on the ZIKV60 aptamer for the differential diagnosis of the Zika virus.

## Introduction

The genus *Flavivirus* comprises 53 virus species that are transmitted by mosquitoes, and the majority of these have the potential to infect humans. Mosquito-transmitted viruses are a significant public health problem in many tropical and sub-tropical countries. Viruses medically important in the genus *Flavivirus* include Zika virus (ZIKV), dengue virus (DENV), and yellow fever virus (YFV), mainly transmitted to humans by *Aedesaegypti* mosquitoes [1–2]. In 1947, ZIKV was first discovered in Uganda in a sentinel monkey [3]. As with other *Flaviviridae* members, ZIKV has a single-stranded RNA genome and three structural proteins, namely capsid, pre-membrane, and envelope, building the capsid, and seven non-structural (NS) proteins, NS1, NS2A, NS2B, NS3, NS4A, NS4B, and NS5, involved in virus replication [4].

The glycoprotein NS1 is a 352-amino-acid polypeptide, which has a molecular weight of 46–55 kDa (depending on its glycosylation status), and is essential in viral replication contributing to different stages of the virus life cycle. The NS1 protein is found at high levels in the blood of infected individuals at initial stages of infection, typically beginning with or before the onset of symptoms. For these reasons, NS1 has a potential diagnostic value as a viral marker of infection [5–7].

Correct diagnosis of a flavivirus infection can be challenging, particularly in world regions where more than one flavivirus co-circulates and YFV vaccination is mandatory. The traditional serological approaches present relatively high antigenic cross-reactivity. Molecular techniques have been successfully applied for flavivirus infection diagnosis offering the advantages of speed, sensitivity, and specific virus identification. However, these techniques require professional expertise and expensive laboratory equipment and reagents. These conditions are frequently unavailable in endemic regions [8–10].

Nucleic acid aptamers are single-strand oligonucleotides (ssDNA or ssRNA) that can bind to other molecules with high affinity and specificity. Aptamers are comparable to antibodies in their applications, yet, they offer several advantages, including higher temperature stability, fast chemical synthesis, easy labeling, versatile chemical modification, flexible structure, and relatively small size. Since aptamers are selected entirely in vitro, toxic compounds as well as molecules with little or no immune response can also serve as targets. Aptamers constitute a class of molecules promising in various applications in health, particularly in diagnosis. In the past two decades, high-affinity nucleic acid aptamers have been developed for a wide variety of targets, such as proteins, peptides, viruses, and bacteria [11–12].

Generally, nucleic acid aptamers are developed in vitro by a molecular evolution process based on iterative selection–amplification steps known as “systematic evolution of ligands by exponential enrichment” (SELEX), first introduced in 1990 [13]. This methodology includes the exposure of the target molecule to a chemically synthesized random-sequence library (10^14^–10^15^ sequences) to allow for the interaction of all binding oligonucleotides to the target. The next step involves the separation of non-bound sequences from sequences that bound to the target for further real-time polymerase chain reaction (PCR) amplification. The enriched pool of aptamers is exposed to the target again. Selection, separation, and amplification steps comprise one SELEX round. Usually, six to as many as 15 rounds have been reported to allow for the selection of the best binders. The PCR products of the last round are cloned into a vector and sequenced for identification of the binding sequences. Over the past three decades, more than 32 SELEX variations have been reported in an attempt to reduce processing time, generate aptamers with novel designs and functions, or increase the process throughput [14–16]. In particular, the CE-SELEX technique is a SELEX variation in which capillary electrophoresis (CE) is used to separate oligonucleotides that have bound to the target [17]. The CE separation efficiency allows for high-affinity aptamers to be obtained with only three to five rounds.

Our present study demonstrates, for the first time, the application of CE-SELEX to obtain DNA aptamers for the ZIKV NS1 protein. In particular, the ZIKV60 aptamer (patent number BR1020210252790), exhibits high binding affinity to NS1 with a *K*_d_ value in the nanomolar range. Also, this ligand showed a higher specificity for ZIKV than for DENV and YFV. Furthermore, we functionalized multiple graphene field-effect transistors with ZIKV60 to demonstrate, by electrical characterization, the selectivity of this aptamer towards ZIKV NS1 protein diluted in human serum. The functionalized graphene devices exhibit an evident recognition of NS1 ranging from 0.01 to 100 pg/mL. As a result, the efficiency of our functionalization protocol in combination with the distinguished specificity and sensibility of ZIKV60 for NS1 may be an innovative tool for novel graphene-based biosensors for ZIKV NS1 protein detection.

## Experimental

### NS1 proteins

Recombinant Zika virus (Uganda strain), dengue virus (serotypes 1, 2, 3, and 4) and yellow fever virus NS1 proteins expressed in mammalian HEK293 cells were purchased from The Native Antigen Company.

### Oligonucleotide library design

The sequence of the 80mer library is described as follows: 5′-CTTCTGCCCGCCTCCTTCC-(39N)-GGAGACGAGATAGGCGGACACT-3′, where the central 39N represents random oligonucleotides based on equal incorporation of A, G, C, and T at each position and was purchased from IDT DNA Technologies [18].

### CE-SELEX

Three CE-SELEX selection cycles were performed. To initiate in vitro selection, 800 μM ssDNA was heated at 95 °C for 5 min, snap-cooled on ice for 15 min and left at room temperature for 10 min. After that, it was incubated with 80 pmol of the ZIKV NS1 protein in borate buffer (60 mM) for 30 min, 200 rpm, at 37 °C. An incubation solution sample was submitted to separation by CE using a neutral capillary on a P/ACE MDQ capillary electrophoresis system (Beckman Coulter Inc., Fullerton, CA). The capillary was first rinsed with 0.1 M HCl for 5 min, water (DNAse/RNAse free) for 1 min, then borate buffer for 5 min under 50 psi pressure before injection of the incubation mixture at 0.5 psi for 5 s. The mixture was separated under 26 kV (reverse polarity) in borate buffer at 25 °C and monitored through UV absorbance detection at 214 nm.

The NS1–aptamer complexes were collected into a vial containing 8 μL of separation buffer at the capillary outlet. After collecting material from five runs, the bound sequences were amplified by PCR. The PCR amplification was performed using a reaction master mix containing 200 μM dNTPs, 0.2 μM forward primer (5′-CTTCTGCCCGCCTCCTTCC-3′), 0.2 μM reverse primer (5′-AGTGTCCGCCTATCTCGTCTCC-3′), 1 U Taq DNA polymerase high fidelity (Invitrogen), and 50 mM MgSO_4_ in 1× high fidelity PCR buffer. The PCR thermocycling parameters were 94 °C for 2 min for denaturation followed by 17 cycles of denaturation at 94 °C for 15 s, annealing at 72 °C for 30 s (−1 °C per cycle) and extension at 68 °C for 30 s. In addition, 14 cycles using denaturation at 94°C for 15 s, annealing at 55 °C for 30 s and extension at 68 °C for 30 s were carried out. A final extension step at 68 °C for 2 min followed the last cycle.

Asymmetric PCR was used to obtain the single-strand DNA of interest from the conventional PCR product. It was achieved using a reaction master mix containing 200 μM dNTPs, 0.2 μM forward primer, 0.02 μM reverse primer, 1 U Taq DNA polymerase high fidelity (Invitrogen), and 50 mM MgSO_4_ in 1× high-fidelity PCR buffer. PCR thermocycling was performed as described above. Conventional and asymmetric PCR reactions were carried out in a Veriti thermal cycler (Applied Biosystems, Foster City, CA). PCR amplification was verified by electrophoresis on 2% agarose gels (in tris–borate–EDTA buffer) stained with ethidium bromide and imaged using a UV transluminator (UVP BioDoc-It Imaging System). The PCR asymmetric product was used to carry out counterselections with NS1 proteins of DENV (serotypes 1, 2, 3, and 4) and YFV. The protocol described by Simmons et al. with the proteins immobilized on Nunc MaxiSorp plates (Thermo Fisher) was used [19]. The sequences unbound to these proteins were collected, amplified by conventional PCR, further amplified by asymmetric PCR, and the obtained ssDNA product was used for the next positive selection cycle with the ZIKV NS1 protein. The counterselections were repeated after each positive selection.

### DNA cloning and sequencing

The ssDNA sequences obtained after the third cycle of the CE-SELEX procedure were cloned and sequenced [20]. PCR was performed, and the products were cloned into pGEM^®^-T easy vector systems (Promega). The plasmid was transformed into *Escherichia coli* DH5α. After electroporation, Luria–Bertani (LB) medium was added and the material was grown for 1 h, at 37 °C, 200 rpm. The culture was then plated onto LB/agar/ampicillin/IPTG/Xgal medium and incubated overnight at 37 °C. The viable white clones were then individually seeded in 10 mL of LB/ampicillin and incubated overnight at 37 °C, 200 rpm. The plasmids were extracted following the manufacturer protocol of the Wizard^®^ Plus SV Minipreps DNA purification system kit (Promega) and quantified via 260 nm absorbance in a spectrophotometer. The vectors were sequenced by the automated Sanger methodology using the M13 promoter primer. The sequencing was carried out at the René Rachou Institute (FIOCRUZ/Minas).

### Secondary structure prediction

The secondary structure was predicted using Mfold analysis [21–22].

### Determination of the dissociation constant

Enzyme-linked oligonucleotide assay (ELONA) was used to determine the dissociation constant *K*_d_ of ZIKV60 [23]. Briefly, NS1 ZIKV (10 pmol) was attached to a Cu^2+^-coated plate overnight. The plate was washed three times in borate buffer. After that, increasing concentrations (1.56, 6.25, 12.5, 25, 50, and 200 nM) of ZIKV60 digoxigenin-labeled ssDNA aptamer (Integrated DNA Technologies) were added in triplicate and incubated at 37 °C. After 30 min of incubation, the plate was washed three times in borate buffer. Aptamers were incubated for 60 min in borate buffer with anti-digoxigenin antibody diluted 1:5000 and labeled with HRP (Roche) following the manufacturer’s instructions. The plate was washed three times in borate buffer, and a colorimetric reaction was initiated by adding 3,30,5,50-tetramethylbenzidine (TMB). After incubating the reaction for 15 min, the reaction was stopped by adding 2 M sulfuric acid. Absorbance at 450 nm was recorded and a saturation curve was plotted. The data were analyzed by non-linear regression analysis (one-site specific binding with Hill slope) using the software GraphPad Prism 5 (Graph-Pad Software, San Diego, CA, USA).

### Specificity assay

In this step, 100 nM of the aptamer in the selection buffer was incubated with 40 pmol of each NS1 protein (DENV serotypes 1, 2, 3, and 4, YFV, or ZIKV) as previously mentioned. The sequences bound and unbound were separated by filtration. The amount of bound sequences in the samples was quantified by real-time quantitative polymerase chain reaction (qPCR) in a Step One real-time PCR System (Applied Biosystems, Foster City, CA) using SYBR green (PowerUp PCR SYBR Green Master Mix, Applied Biosystems). A standard curve for each aptamer was recorded ranging from 0.05 to 0.00005 ng with a dilution factor of 1:10. All samples and negative and positive controls were processed in triplicate. The mass of aptamers bound to each protein was calculated and the results were plotted using the software Graph Pad Prism 5. Statistical analysis was performed using the Kruskal–Wallis test with Dunn’s post-test.

### Device fabrication and electrical characterization

We carried out the electrical characterization utilizing field-effect transistors fabricated using single-layer graphene grown by chemical vapor deposition (CVD) and transferred to Si/SiO_2_ substrates. The wafers were purchased from Graphene Platform and we produced graphene transistors by conventional photolithography, following the procedures previously described by our group elsewhere [24]. The dimensions of the sensing region (100 μm × 100 μm) were defined by O_2_ plasma etching. The source and drain electrodes were fabricated by electron-beam evaporation of 2 nm/100 nm of Ti/Au, and we covered the metal contacts with a 10 μm thick passivation layer of SU-8. Figure S1a (Supporting Information File 1) shows an optical microscopy image of the typical CVD graphene device used in this work.

The functionalization of the graphene devices with ZIKV60 aptamers was realized by overnight incubation in wet atmosphere with a 5 μL drop of 1 μM ZIKV60 solution dispersed in 1 mM PBS (phosphate-buffered saline: 0.0001 M phosphate buffer, 0.000027 M potassium chloride, and 0.00137 M sodium chloride), pH 7.4. After incubation, the graphene devices were rinsed with 1 mM PBS, followed by sequential rinsing with deionized water and drying in a nitrogen flow.

The electrical characterization for both demonstration of graphene functionalization with ZIKV60 aptamers and ZIKV NS1 protein detection consisted of DC measurements of the graphene transistors transfer characteristics. We conducted these measurements via electrolyte gating, utilizing a Keysight B2902A Precision Source/Measure Unit, immediately after the functionalization process. The source–drain bias was fixed at 1 mV, and we applied the gate voltage via a gold electrode in contact with 100 mM PBS or human serum. PBS was used as electrolyte for validation of the graphene functionalization with ZIKV60 aptamers, while human serum was adopted for ZIKV NS1 protein detection. Human serum (from human male AB plasma) was obtained from Sigma-Aldrich. We stress the fact that by using human serum as the gating electrolyte we can directly address any question about the selectivity of the detection since the human serum already contains a plethora of interfering biomolecules [25]. Figure S1b (Supporting Information File 1) exhibits a schematic illustration of the resulting ZIKV60-functionalized graphene devices and the experimental setup used in the electrical characterization for ZIKV NS1 protein detection.

## Results and Discussion

A simple, economical, sensitive, and specific diagnostic test is required to help to control flavivirus infections. The serological approaches currently available are limited due to the cross-reactivity between the members of the *Flavivirus* genus. Molecular diagnosis, although effective, requires expensive laboratory facilities frequently unavailable in endemic regions. Therefore, it is necessary to develop new technologies able to distinguish with precision the medically important viruses of this genus. The aim of the present study was to obtain DNA aptamers for the ZIKV NS1 protein, capable of providing a specific virus identification for application in diagnostic biosensors. In this context, biosensor platforms based on aptamers (aptasensors) are commonly used to assess and quantify in real-time, with high sensitivity, the presence of an analyte, such as a protein [26]. Aptasensors for hepatitis C, H5N1 avian influenza, and H1N1 viruses, among others, have been developed [27–28]. Furthermore, aptamers have the potential to overcome the lacking functional and storage stability of most biosensors exploiting antibodies.

**Figure 1:**
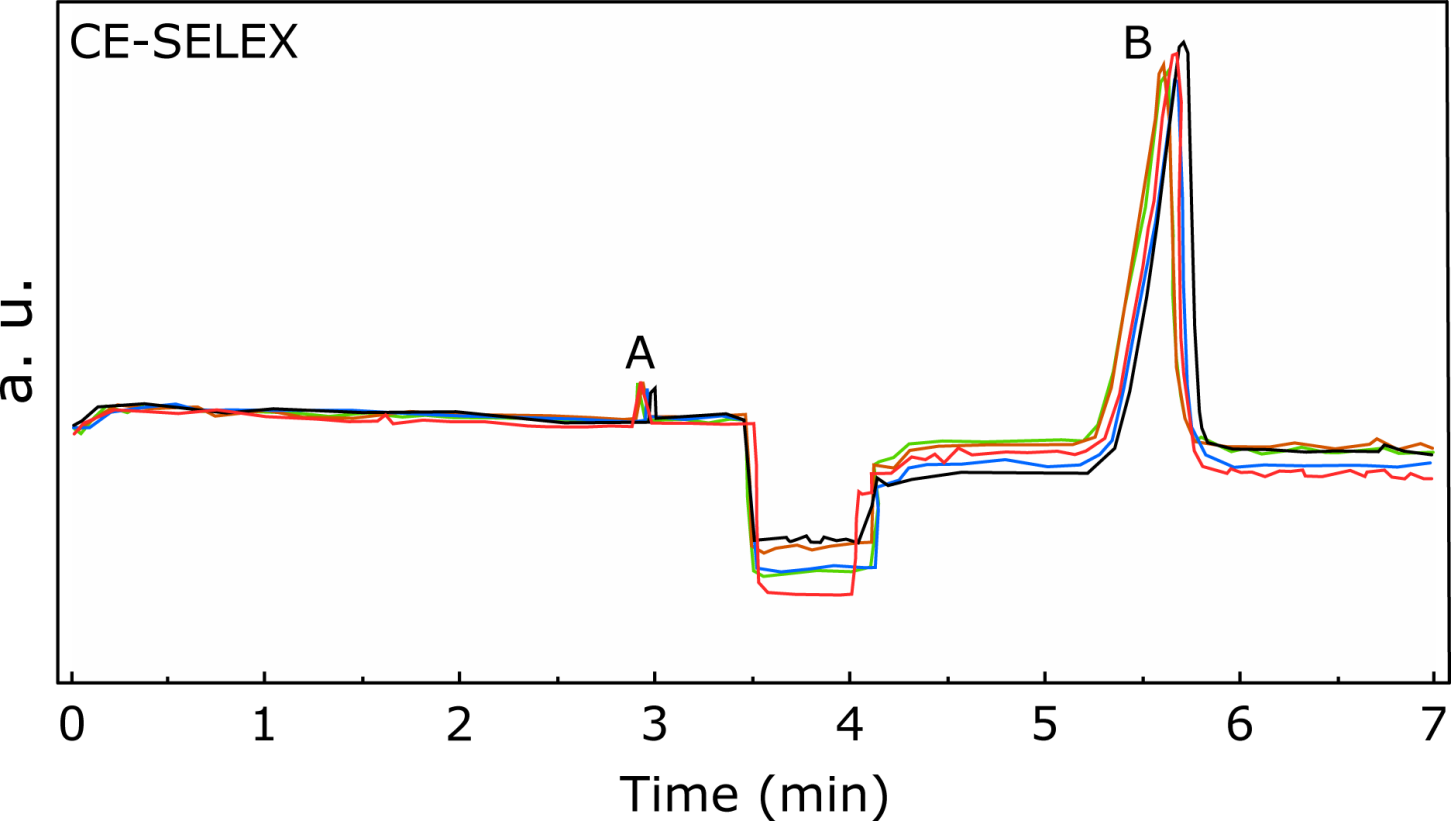
Representative CE electropherogram of CE-SELEX separation step. Peak A corresponds to the NS1–aptamers complexes, while peak B refers to oligonucleotides that did not bind to the target and were discarded. Five runs, which are displayed by distinctly colored lines, were performed to accumulate enough DNA for PCR amplification. These runs are superimposed in the graph.

In the present work, CE-SELEX was used for aptamer selection. This procedure uses CE in the SELEX separation step. A representative CE electropherogram is shown in Figure 1. The elution peaks corresponding to the NS1–aptamer complexes and the oligonucleotides that did not bind to the target occurred at different positions. Five runs were performed to accumulate enough DNA for the PCR amplification step and these runs are superimposed in Figure 1. Counterselections with NS1 proteins of DENV (serotypes 1, 2, 3, and 4) and YFV were performed after each positive selection with ZIKV NS1 protein, as described in the CE-SELEX selection schematic diagram of Figure S2 (Supporting Information File 1) as well as in the Experimental section.

After the third selection cycle, the obtained ssDNA pools were cloned and sequenced. Seventy-five sequences were identified. The most prevalent sequences were chosen for further characterization. Among these, the sequence ZIKV60 presented the best results in terms of affinity and specificity. Figure S3 (Supporting Information File 1) illustrates the secondary structure of ZIKV60 aptamer predicted by Mfold software.

**Figure 2:**
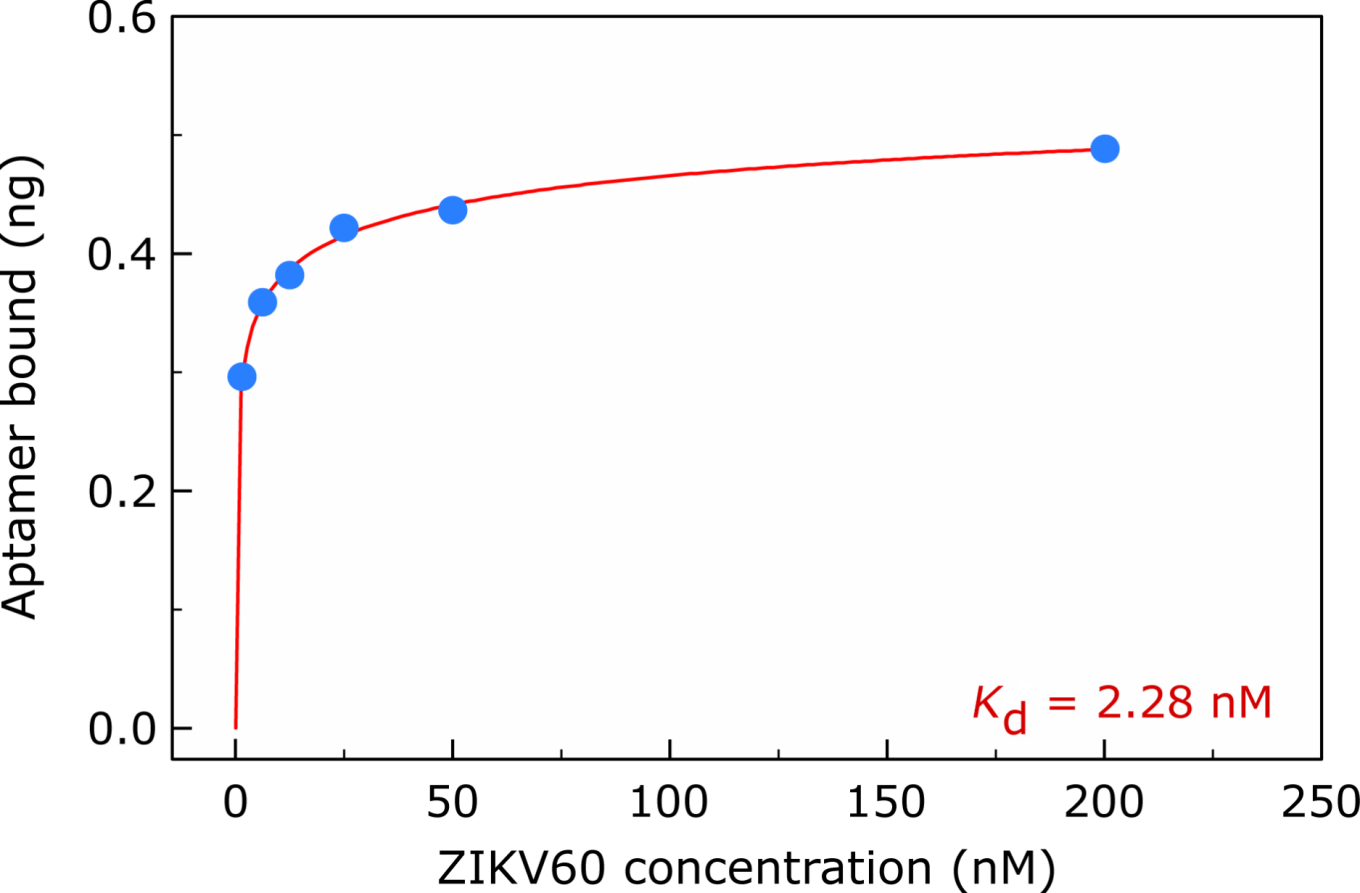
Dissociation constant (*K*_d_) of the ZIKV60 aptamer measured by ELONA. The saturation curve was obtained by plotting the aptamer concentration as a function of the total mass of ssDNA bound to ZIKV NS1. The value of *K*_d_ was calculated by non-linear regression analysis (one-site specific binding with Hill slope, software GraphPad Prism 5), resulting in *K*_d_ = 2.28 ± 0.28 nM (R^2^ = 0.9958).

The aptamer affinity to the target is indicated by the dissociation constant at equilibrium (*K*_d_). For the determination of *K*_d_ of the aptamers, a saturation-binding curve to the target was measured with an increasing series of ZIKV60 concentrations. The aptamer quantification was performed by ELONA [23]. ZIKV60 presented a high binding affinity to ZIKV NS1 protein with a *K*_d_ value of 2.28 ± 0.28 nM. Figure 2 illustrates the ZIKV60 aptamer saturation binding curve to ZIKV NS1.

**Figure 3:**
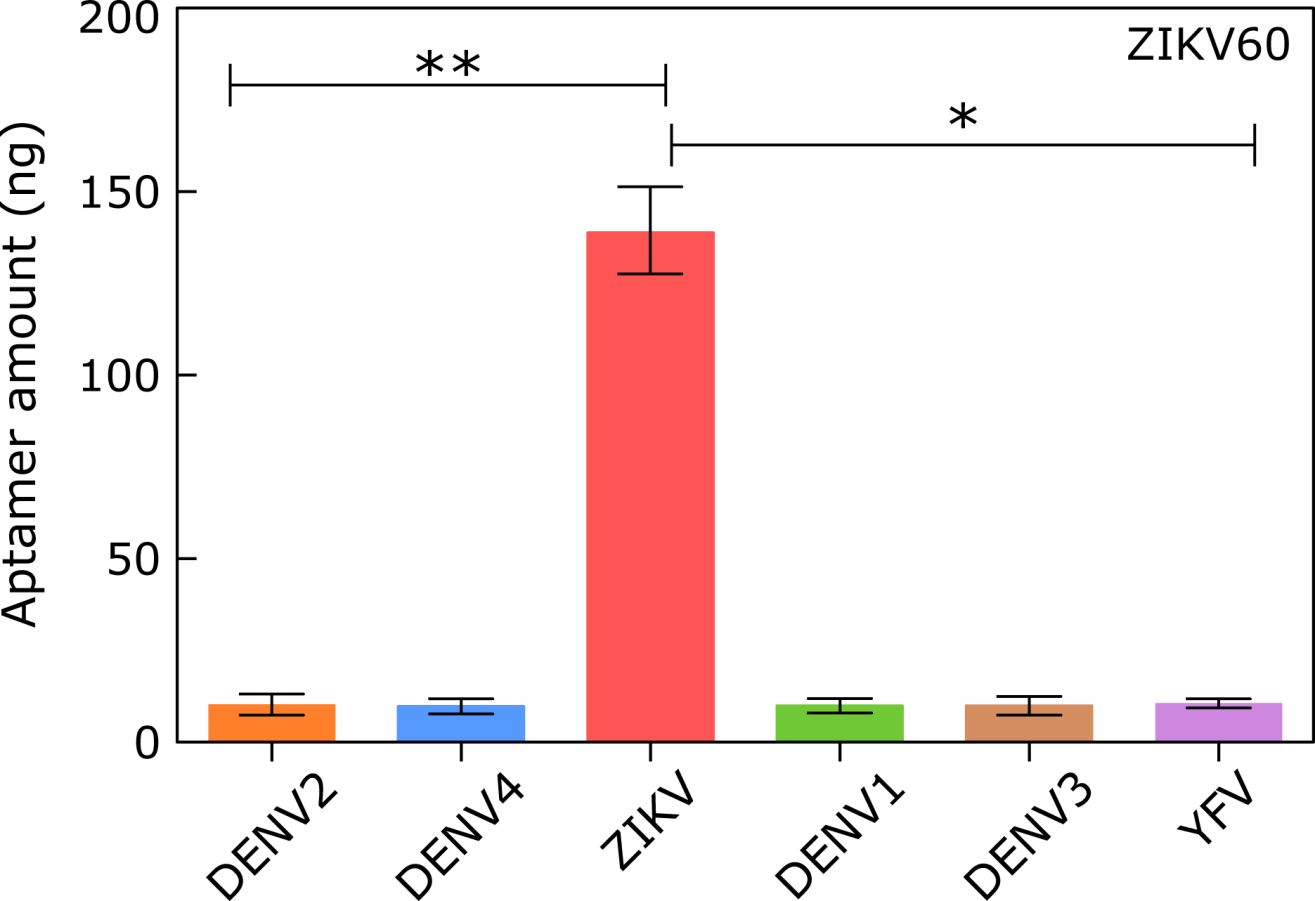
Binding of ZIKV60 aptamers to NS1 protein of different flaviviruses performed by qPCR. The graphs show the ssDNA amount (ng) recovered after incubation of 100 nM of ZIKV60 with NS1 protein (40 pmol) of the different flaviviruses: ZIKV, DENV (serotypes 1, 2, 3, and 4), and YFV. Statistical analyses were performed using the GraphPad Prism 5 software. ^*^p < 0.05 and ^**^p < 0.01.

The aptamer specificity was evaluated by qPCR [29–33]. The ZIKV60 binding to the NS1 protein of DENV (serotypes 1, 2, 3, and 4) and YFV was also determined. The results are shown in Figure 3. The specificity assay confirmed that ZIKV60 binds preferentially to the NS1 ZIKV protein relative to NS1 of the other viruses, with the following ratios: DENV1 14.2, DENV2 14.8, DENV3 13.4, DENV4 14.8, and YFV 14.2 (Table 1).

**Table 1:** ZIKV60 aptamer characteristics.

Aptamer	Δ*G* (kcal/mol)	*K*_d_ (nM)	Target/non-target ratio^a^

ZIKV60	−21.75	2.28 ± 0.28	DENV1: 14.2
			DENV2: 14.8
			DENV3: 13.4
			DENV4: 14.8
			YFV: 14.2

^a^Ratio between the binding of ZIKV60 to the NS1 protein of the Zika virus in comparison to the NS1 protein of the other tested flaviviruses

Applying CE to SELEX (CE-SELEX) was an important advance. The separation efficiency of CE contributed to the reduction of the number of cycles necessary for the selection of high-affinity aptamers. As the molecules remain in solution during the separation process, there is no steric hindrance for aptamers binding to the target [26]. In addition, the native conformation of the protein targets is retained and is the same as found in biological samples. To obtain aptamers specific for the ZIKV NS1 protein, counterselections with NS1 proteins of DENV (serotypes 1, 2, 3, and 4) and YFV were included. Carrying out counterselections with homologous proteins is advantageous to avoid cross-reactivity. By this approach, it was possible to obtain the ZIKV60 aptamer that, in addition to the high affinity (*K*_d_ = 2.28 ± 0.28 nM), showed a high specificity for ZIKV NS1. The ZIKV60 aptamer binding to ZIKV NS1 protein was around 14 times higher than the binding to NS1 proteins of the other flavivirus.

According to the selectivity assay results, the specificity of ZIKV60 to ZIKV NS1 protein is considerably higher than that to NS1 of the other viruses. Concerning the development of aptasensors for ZIKV NS1 protein detection, the results show that the biosensors based on ZIKV60 aptamer would yield improved protein recognition. Following this concept, we functionalized multiple graphene field-effect transistor devices with ZIKV60 aptamers to demonstrate the feasibility of constructing graphene-based aptasensors for ZIKV NS1 protein detection. Importantly, these ZIKV60 aptamers feature a pyrene moiety for their direct immobilization on graphene via π–π stacking [34].

**Figure 4:**
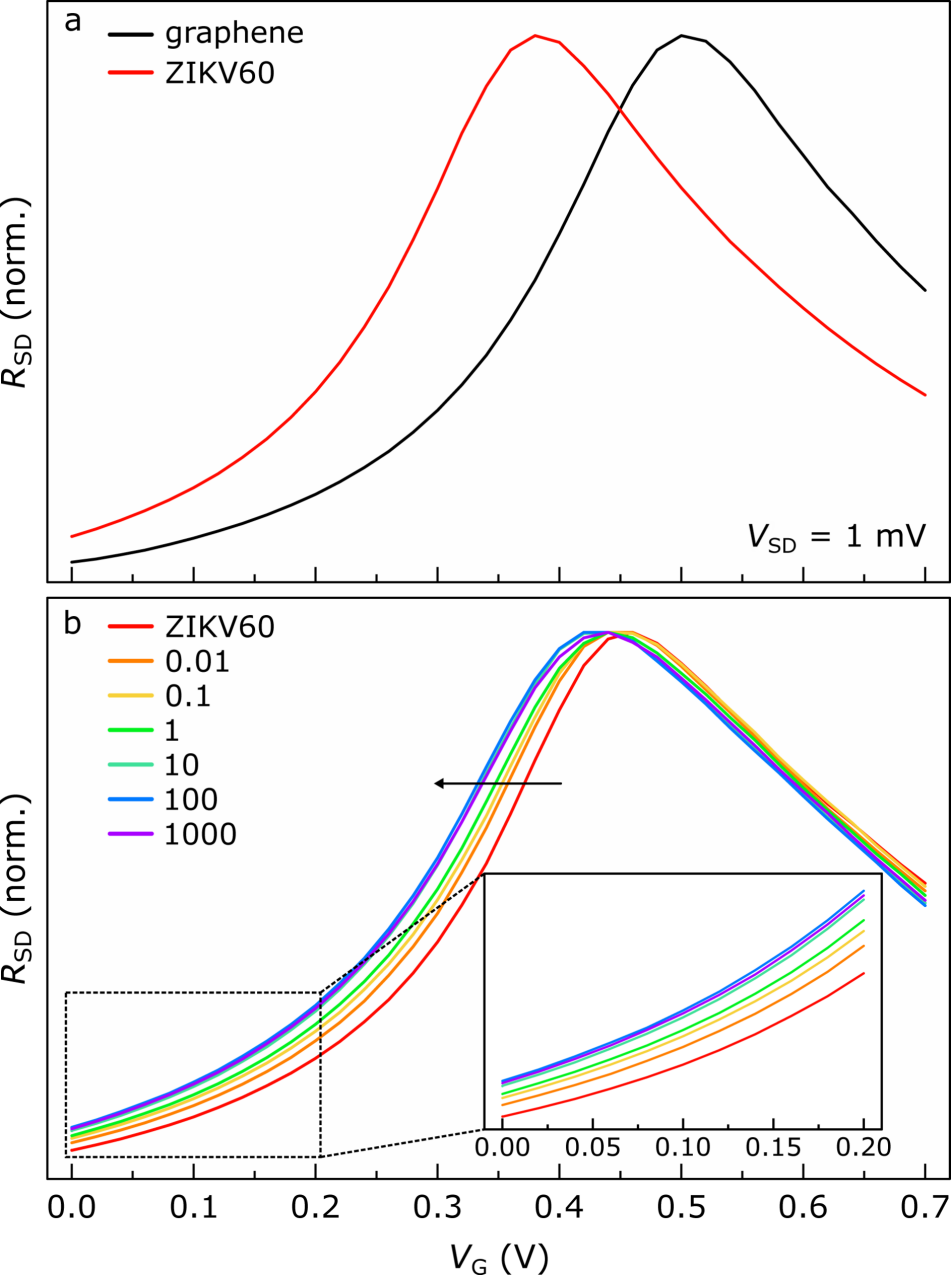
Functionalization of graphene with ZIKV60 and detection of ZIKV NS1 protein. (a) Representative curves of the normalized graphene resistance (*R*_SD_) as a function of the gate voltage (*V*_G_) before (black line) and after (red line) functionalization with ZIKV60 aptamers. These measurements were carried out using 100 mM PBS as electrolyte for gating. (b) Normalized graphene transfer curves of a single device functionalized with ZIKV60 aptamers before (red line) and after sequential addition of ZIKV NS1 protein with the following concentrations: 0.01 (orange line), 0.1 (yellow line), 1 (green line), 10 (cyan line), 100 (blue line), and 1000 (purple line) pg/mL. The inset highlights the portion of each graphene transfer curve extending from 0.00 to 0.20 V. In each measurement, ZIKV NS1 protein was diluted in human serum, which was used as electrolyte for gating.

The qualitative confirmation of aptamer immobilization on graphene is illustrated in Figure 4a. For a representative device, Figure 4a exhibits curves of graphene resistance (*R*_SD_) as a function of the gate voltage (*V*_G_) before (black line) and after (red line) functionalization with ZIKV60 aptamers. In this experiment, we used 100 mM PBS as electrolyte for gating. The π-conjugated units of the pyrene-modified ZIKV60 aptamers transfer electrons to graphene, resulting in the left-shift of the graphene transfer curve because of the aptamer adsorption. Several studies support this electron transfer from pyrene-modified molecules to graphene as the binding mechanism in π–π interactions between such compounds [35–37]. The association of pyrene-modified ZIKV60 aptamers with graphene may also be mediated by charge transfer that assists the interaction between the pyrene moiety of ZIKV60 and the π orbitals of graphene [38–39]. Consequently, the electron transfer to graphene after functionalization reveals a factual immobilization of ZIKV60 aptamers on its surface. Similar results were obtained for four additional graphene devices. See Figure S4 (Supporting Information File 1) for more details.

In the following, we address the capability of ZIKV60-functionalized graphene devices to detect distinctively the ZIKV NS1 protein. We conducted electrical measurements of graphene transfer curves to evaluate the binding of ZIKV NS1 diluted in human serum to ZIKV60 ligands. The response of a representative ZIKV60-functionalized graphene device to the sequential addition of increasing amounts of NS1, from 0.01 up to 1000 pg/mL, are shown in Figure 4b. For each specific concentration, we first exposed the graphene device to the electrolyte containing the NS1 protein for 10 min. Then, we measured the corresponding electrical response. The characterization of ZIKV60 alone (red line in Figure 4b) was carried out after 10 min of incubation with human serum not containing NS1 to provide a basis for comparison to upcoming receptor–target conjugations occurring in protein-rich environments. From 0.01 to 100 pg/mL, the graphene transfer curve left-shifts successively as a result of progressive additions of five specific protein dilutions. This denotes a cumulative electron transfer to graphene as NS1 binds to ZIKV60. However, this trend is interrupted at the cutoff value of 100 pg/mL. In fact, the inset in Figure 4b evidences only minimal alterations of the graphene transfer curve corresponding to 100 pg/mL (blue line) after adding 1000 pg/mL of NS1 (purple line). Physically, this outcome is sustained by a complete saturation of the active ZIKV60 binding sites with NS1 due to exposure to protein dilutions of higher concentrations, such as 100 pg/mL. Consequently, further protein incorporation results in inappreciable specific recognition as well as negligible charge transfer to graphene.

**Figure 5:**
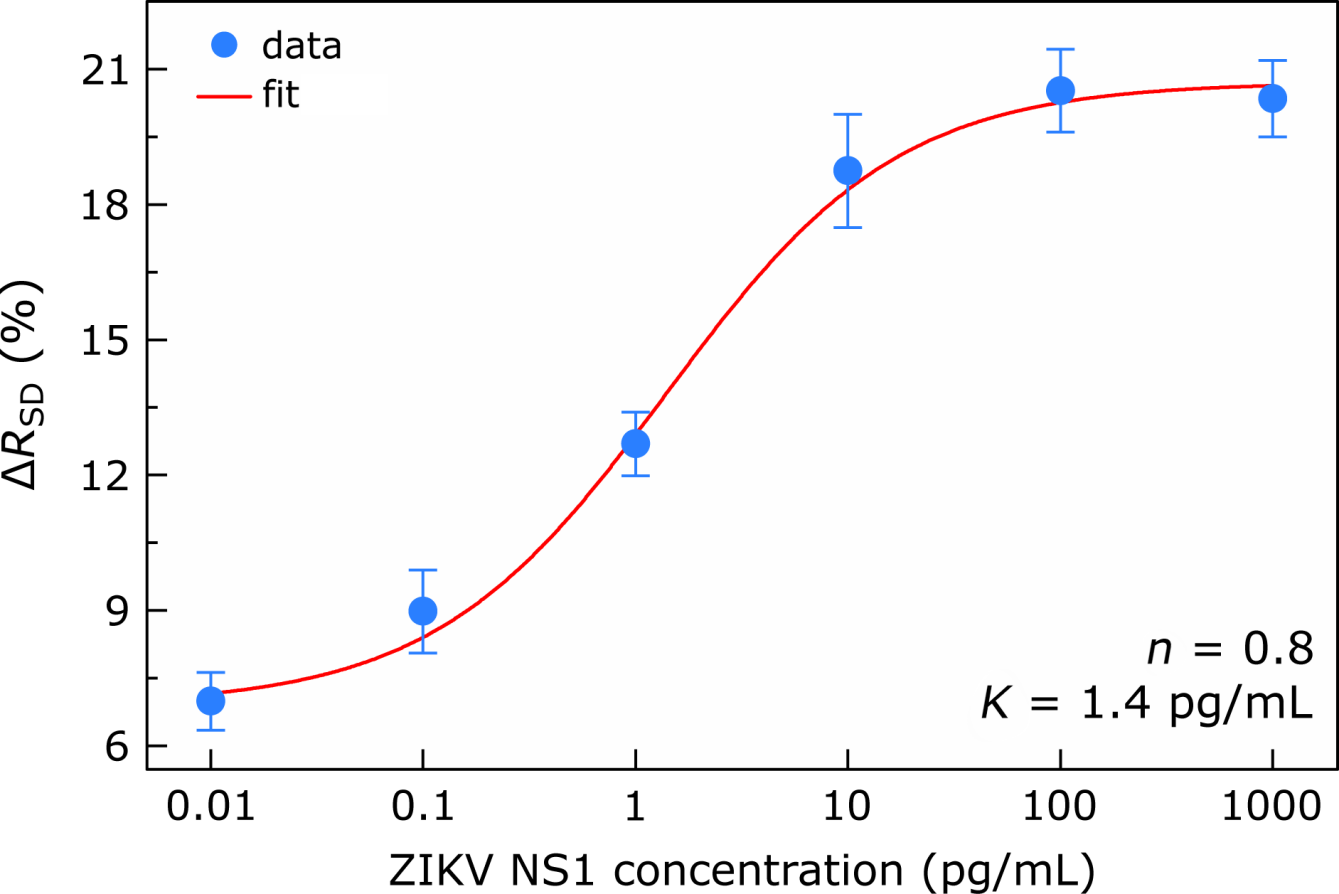
Calibration curve for ZIKV NS1 protein detection. Percentage change in graphene resistance (Δ*R*_SD_) at *V*_G_ = 0.2 V as a function of the ZIKV NS1 protein concentration. For each specific dilution, the blue filled circle represents the mean percentage increment in graphene resistance accounted for seven different devices; the error bars show the standard error of the measurements. The red line is a Hill–Langmuir curve fitted to the experimental data (R^2^ = 0.9969).

This observation has been made consistently with six additional graphene devices, whose detection range extends from 0.1 to 100 pg/mL, see Figure S5 (Supporting Information File 1) for more details. We therefore analyzed in depth the general dose-dependent responses of all seven aptasensors. Figure 5a exhibits a calibration curve for ZIKV NS1 detection, reflecting the average behavior of this group of sensing devices with respect to protein addition. We evaluated the sensor responses as percentage changes in graphene resistance (Δ*R*_SD_) at *V*_G_ = 0.2 V caused by aptamer–protein binding. For more details on how the variations in graphene resistances were measured, see Table S1 and Table S2 (Supporting Information File 1). For each specific ZIKV NS1 dilution, the blue filled circle in Figure 5 represents the mean percentage increment in graphene resistance for the seven different devices, while the error bars give the standard error of the measurements. The pattern that emerges from Figure 5 clearly reveals that the collective response of the graphene sensors as a function of the ZIKV NS1 concentration follows a sigmoidal trend. Given that, we interpolated a Hill–Langmuir curve, Δ*R*_%_(*c*) ∝ *c^n^* (*k^n^* + *c^n^*)^−1^, to the experimental data. This fit curve is represented by the red line Figure 5 [40]. The percentage changes in graphene resistance, Δ*R*_%_, as a function of the ZIKV NS1 concentration, *c*, yielded a microscopic dissociation constant *k* = 1.4 ± 0.3 pg/mL and a Hill coefficient of *n* = 0.8 ± 0.2. The microscopic dissociation constant provides an estimate for the extent of target molecules producing half-occupation on ligand binding sites. Correspondingly, the value obtained for *k* corroborates the hypothesis that our aptasensors are fully capable of selective and sensitive recognition of ZIKV NS1 protein at picogram per milliliter concentrations.

The levels of NS1 in individuals infected with Zika virus are not completely clear yet. However, Yap et al. recently reported that the normal values for ZIKV NS1 antigen found in clinical samples during acute infection are extremely low or undetectable by ELISA in most cases [41]. In that report, the authors verified that the degree of ZIKV NS1 in 60% of the ill patients was lower than 0.1 ng/mL, which was the limit of detection of their assay. In contrast, the platform reported here surpassed considerably the limitations of ELISA concerning the detection of ZIKV NS1 antigen. Our aptasensors recognized significantly small protein quantities, as low as 0.01 pg/mL, coexisting with large amounts of blood components. For comparison, Afsahi et al. described a biosensor based on graphene utilizing monoclonal antibodies as recognition elements for ZIKV NS1 that could detect 500 ng/mL of the target protein diluted in human serum [42]. Therefore, the potential of ZIKV60 aptamer in association with graphene to detect the NS1 protein of Zika virus on a clinical relevant scale offers the implementation of this hybrid system for early diagnosis of ZIKV infection in real samples in the future.

Aptamers for ZIKV NS1 protein have been reported in the literature in only two other studies so far. Lee and Zeng selected an aptamer (APT2) with high affinity (*K*_d_ = 24 pM) but exhibiting cross-reactivity for the NS1 protein of all DENV serotypes. They also obtained a second aptamer (APT10) with negligible cross-reactivity, but less affinity (*K*_d_ = 134 nM) [43]. In another study, Morais et al. performed a DNA aptamer selection by immobilizing ZIKV NS1 on ELISA plates [44]. Seven selection cycles were used, including a counterselection step for YFV NS1 in the last cycle. Nonetheless, data concerning aptamer affinity and specificity were not reported in that study. In the present study, an aptamer that combines high affinity and specificity to ZIKV NS1 was successfully obtained. The binding capacity of ZIKV60 to the target protein was exhaustively demonstrated using different methods, namely ELONA (*K*_d_ determination assay), qPCR (specificity assay), and biosensing with functionalized graphene devices. To the best of our knowledge, our work is the first to demonstrate aptamer selection for ZIKV NS1 and, simultaneously, its application on a biosensing platform, creating possible routes for the future development of point-of-care diagnostic systems for Zika virus disease.

## Conclusion

This is the first work describing the use of CE-SELEX for ZIKV NS1 protein aptamer selection. An aptamer suitable for ZIKV differential diagnosis was obtained, which combines high binding affinity for ZIKV NS1 with high specificity relative to the NS1 proteins of the four DENV serotypes and YFV. These attributes make ZIKV60 aptamer superior to other ZIKV NS1 aptamers reported so far. In addition, we validated the combination of ZIKV60 aptamer with FET devices based on graphene as a platform to assist the construction of highly sensitive aptasensors for ZIKV NS1 protein detection.

## Supporting Information

Optical microscopy image of our typical CVD graphene device, schematic illustration of the resulting ZIKV60-functionalized graphene devices and experimental setup used in the electrical characterization for ZIKV NS1 protein detection, flowchart outlining the execution of CE-SELEX for ZIKV NS1 protein aptamers selection, secondary structure of ZIKV60 aptamer, transfer curves of four distinct graphene devices prior and subsequent to functionalization with ZIKV60 aptamer, transfer curves of six individual graphene devices functionalized with ZIKV60 aptamer before and after sequential addition of ZIKV NS1 protein ranging from 0.01 to 1000 pg/mL, graphene resistances at *V*_G_ = 0.2 V of seven different devices for the following ZIKV NS1 protein concentrations: 0.01, 0.1, 1, 10, 100, and 1000 pg/mL, percentage changes in graphene resistance at *V*_G_ = 0.2 V for the following ZIKV NS1 protein concentrations: 0.01, 0.1, 1, 10, 100, and 1000 pg/mL.

File 1 Additional experimental data.
