# Supplementary material for: DNA aptamer selection and construction of an aptasensor based on graphene FETs for Zika virus NS1 protein detection
Source: Beilstein J Nanotechnol. 2022 Sep 2;13:873–81. doi: 10.3762/bjnano.13.78 (PMC9443353; doi:10.3762/bjnano.13.78)
Supplement: File 1 — Additional experimental data. [file Beilstein_J_Nanotechnol-13-873-s001.pdf]

BEILSTEIN JOURNAL OF NANOTECHNOLOGY

Supporting Information

for

DNA aptamer selection and construction of an aptasensor based on graphene FETs for Zika virus NS1 protein detection

Nathalie B. F. Almeida, Thiago A. S. L. Sousa, Viviane C. F. Santos, Camila M. S. Lacerda, Thais G. Silva, Rafaella F. Q. Grenfell, Flavio Plentz and Antero S. R. Andrade

Beilstein J. Nanotechnol. **2022,** *13,* 873–881. doi:10.3762/bjnano.13.78

Additional experimental data

License and Terms: This is a supporting information file under the terms of the Creative Commons Attribution License (https://creativecommons.org/licenses/by/4.0). Please note that the reuse, redistribution and reproduction in particular requires that the author(s) and source are credited and that individual graphics may be subject to special legal provisions.
The license is subject to the *Beilstein Journal of Nanotechnology* terms and conditions: (https://www.beilstein-journals.org/bjnano/terms)

Contents:

1. Optical microscopy image of our typical CVD graphene device, schematic illustration of the resulting ZIKV60-functionalized graphene devices and measurement setup used in the electrical characterization for ZIKV NS1 protein detection (p. S3)

2. Flowchart outlining the execution of CE-SELEX for ZIKV NS1 protein aptamers selection (p. S4)

3. Secondary structure of the ZIKV60 aptamer (p. S5)

4. Transfer curves of four distinct graphene devices prior and subsequent to functionalization with ZIKV60 aptamers. (p. S6)

5. Transfer curves of six individual graphene devices functionalized with ZIKV60 aptamers before and after sequential addition of ZIKV NS1 protein ranging from 0.1 to 1000 pg/mL (p. S7)

6. Graphene resistance at V_G = 0.2 V of seven different devices for the following ZIKV NS1 protein concentrations: 0.01, 0.1, 1, 10, 100, and 1000 pg/mL. (p. S8)

7. Percentage changes in graphene resistance at V_G = 0.2 V for the following ZIKV NS1 protein concentrations: 0.01, 0.1, 1, 10, 100, and 1000 pg/mL (p. S9)

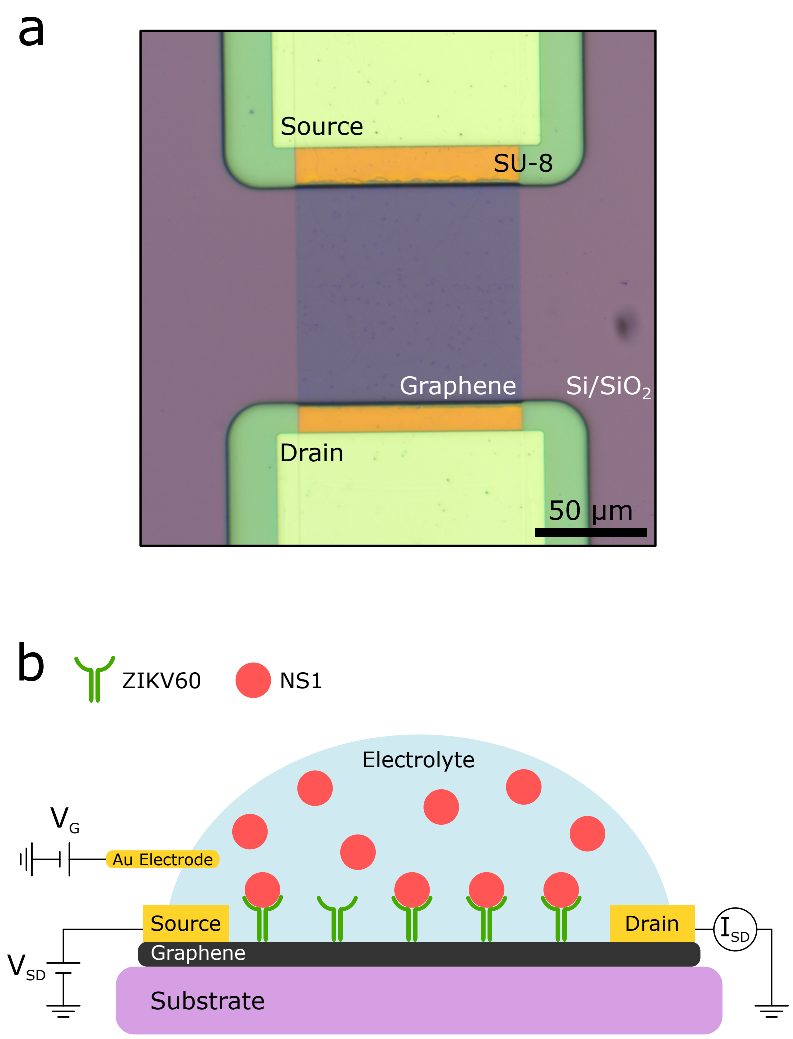

Figure S1: (a) Optical microscopy image of our typical CVD graphene device. (b) Schematic illustration of the resulting ZIKV60-functionalized graphene devices and experimental setup used in the electrical characterization for NS1 protein detection. In this case, human serum was employed as electrolyte to apply the gate voltage.

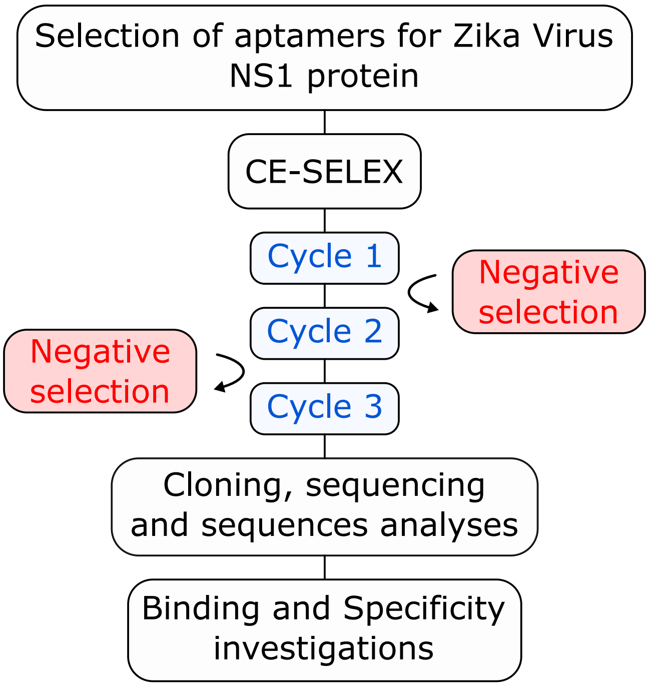

Figure S2: Schematic diagram of the CE-SELEX methodology utilized for ZIKV NS1 protein aptamer selection. The flowchart illustrates the execution of three CE-SELEX cycles. Counter selections with NS1 proteins of DENV (serotypes 1, 2, 3, and 4) and YFV were performed after each positive selection with ZIKV NS1 protein. After the third selection cycle, the ssDNA pool was cloned and sequenced. Then, all sequences were analyzed by bioinformatics to determine their secondary structure and ΔG. The chosen sequences were characterized in terms of affinity for the target (K_d) and specificity for NS1 protein of ZIKV.

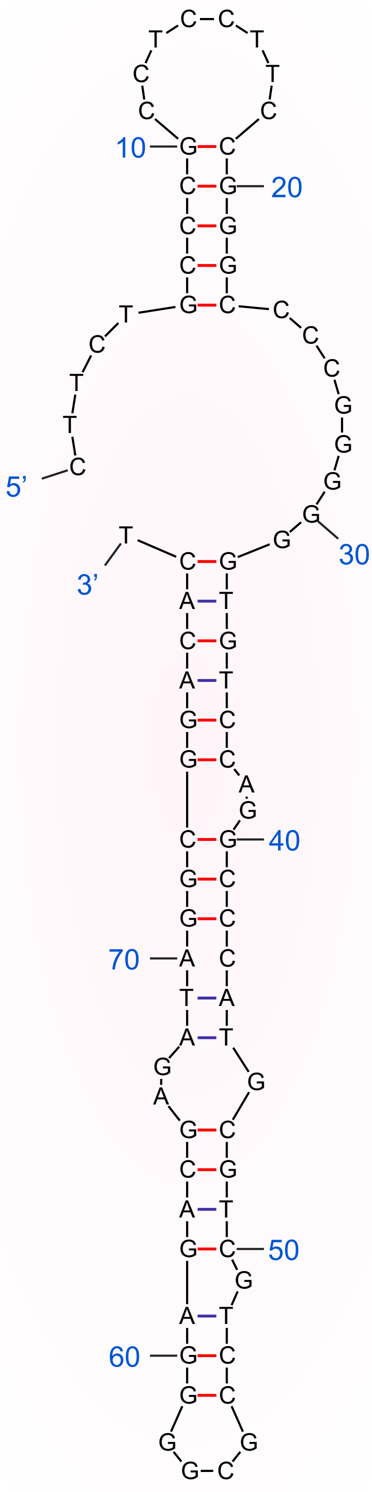

Figure S3: Secondary structure of the ZIKV60 aptamer predicted by Mfold ($\Delta G = -21.75$ kcal/mol).

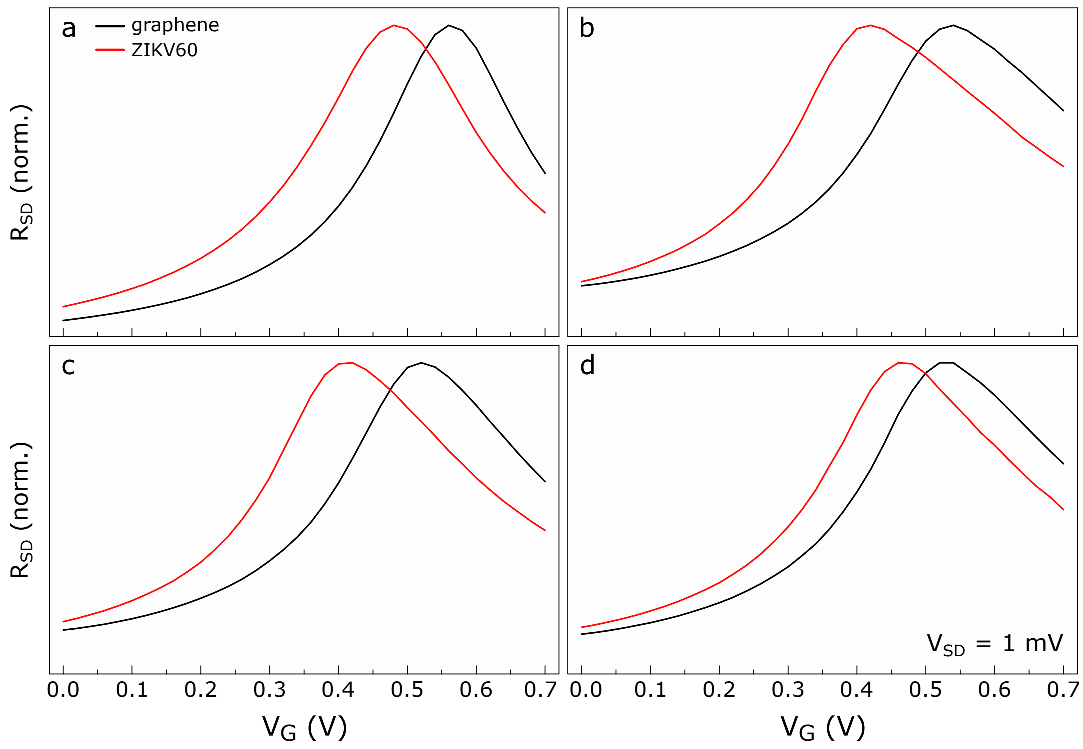

Figure S4: (a–d) Normalized graphene resistance (R_{SD}) as a function of the gate voltage (V_G) of four distinct graphene devices prior (black lines) and subsequent to (red lines) functionalization with ZIKV60 aptamers. These measurements were carried out using 100 mM PBS as electrolyte for gating.

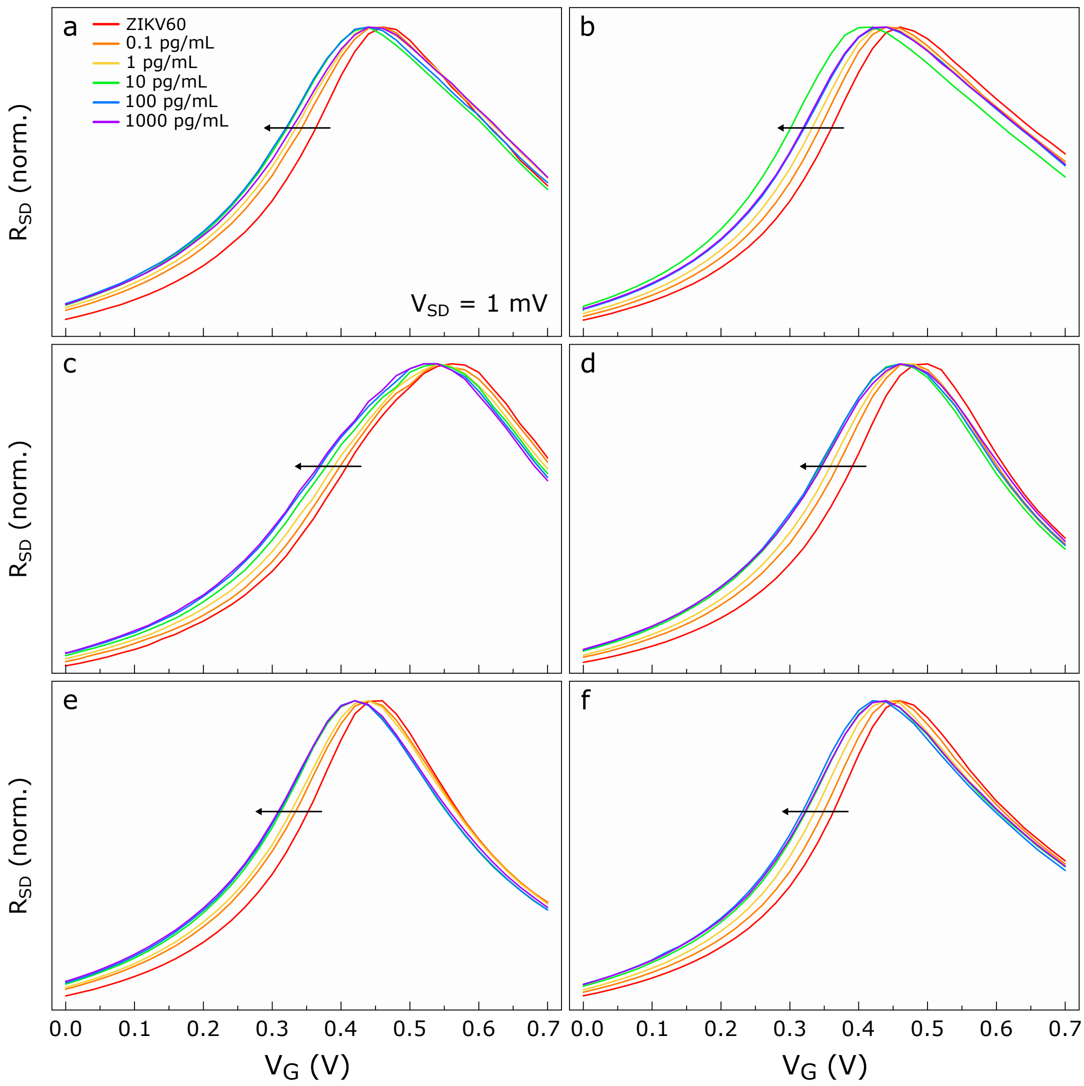

Figure S5: a–f) Normalized graphene resistance (R_{SD}) as a function of the gate voltage (V_G) of six individual graphene devices functionalized with ZIKV60 aptamers before (red lines) and subsequent to sequential addition of ZIKV NS1 protein with the following concentrations: 0.1 (orange lines), 1 (yellow lines), 10 (green lines), 100 (blue lines), and 1000 (purple lines) pg/mL. In each measurement, ZIKV NS1 protein was diluted in human serum, which was used as electrolyte for gating.

Table S1: Graphene resistance at V_G = 0.2 V of seven different devices after exposures to each following amount of ZIKV NS1: 0.01, 0.1, 1, 10, 100, and 1000 pg/mL. For each device, its respective ZIKV60 (aptamer alone) attribute corresponds to the scenario preceding protein additions and this value is used as a reference for estimations of percentage changes in graphene resistance accounted for NS1 incorporations.

ZIKV NS1 concentration (pg/mL)	Device 1 (kΩ)	Device 2 (kΩ)	Device 3 (kΩ)	Device 4 (kΩ)	Device 5 (kΩ)	Device 6 (kΩ)	Device 7 (kΩ)
ZIKV60	6.19	11.22	6.61	4.97	10.75	8.77	5.89
0.01	6.58	12.15	7.03	—	—	—	—
0.1	6.79	12.45	7.29	5.30	11.14	9.71	6.22
1	6.99	12.85	7.50	5.56	11.79	10.09	6.55
10	7.26	13.55	7.92	6.26	12.29	10.79	6.92
100	7.38	13.66	8.05	5.88	12.64	10.94	7.08
1000	7.35	13.37	8.12	5.93	12.80	10.91	7.02

Correspondence of devices:

- **Device 1**: device exhibited in the main text
- **Device 2**: device exhibited in Figure S5a
- **Device 3**: device exhibited in Figure S5e
- **Device 4**: device exhibited in Figure S5b
- **Device 5**: device exhibited in Figure S5c
- **Device 6**: device exhibited in Figure S5d
- **Device 7**: device exhibited in Figure S5f

Table S2: Percentage changes in graphene resistance (ΔR_{SD}) at V_G = 0.2 V of seven individual devices accounted for additions of the following extents of ZIKV NS1: 0.01, 0.1, 1, 10, 100, and 1000 pg/mL. For every amount of NS1, the percentage increment in graphene resistance of each single device was evaluated relative to its condition prior to exposures to ZIKV NS1 (aptamer alone).

ΔR_{SD} (%)	0.01 pg/mL	0.1 pg/mL	1 pg/mL	10 pg/mL	100 pg/mL	1000 pg/mL
Device 1	6.32	9.64	12.86	17.21	19.19	18.65
Device 2	8.26	10.95	14.53	20.73	21.72	19.11
Device 3	6.38	10.27	13.49	19.88	21.81	22.80
Device 4	—	6.67	12.01	26.03*	18.46	19.33
Device 5	—	3.56	9.66	14.25	17.57	19.07
Device 6	—	10.68	15.04	22.98	24.73	24.32
Device 7	—	5.66	11.26	17.43	20.17	19.16
Mean Value	6.99	8.98	12.69	18.75	20.52	20.35
Standard Error	1.11	2.24	1.88	3.08	2.44	2.25

*This element was not included in the estimation of both mean value and standard error corresponding to 10 pg/mL because this particular feature differs significantly from the observations related to the other six devices (please see the green line shown in Figure S5b).

Correspondence of devices:

- **Device 1**: device exhibited in the main text
- **Device 2**: device exhibited in Figure S5a
- **Device 3**: device exhibited in Figure S5e
- **Device 4**: device exhibited in Figure S5b
- **Device 5**: device exhibited in Figure S5c
- **Device 6**: device exhibited in Figure S5d
- **Device 7**: device exhibited in Figure S5f
